# The Outcomes of Trauma-Informed Practice in Youth Justice: An Umbrella Review

**DOI:** 10.1007/s40653-024-00634-5

**Published:** 2024-04-22

**Authors:** Catia G. Malvaso, Andrew Day, Carolyn M. Boyd

**Affiliations:** 1 School of Psychology, The University of Adelaide, Adelaide, South Australia Australia; 2 School of Public Health, The University of Adelaide, Adelaide, South Australia Australia; 3 School of Social and Political Sciences, The University of Melbourne, Parkville, Victoria Australia; 4 Swinburne University of Technology, Hawthorn, Victoria Australia

**Keywords:** Trauma-informed, Trauma-focused, Youth justice, Outcomes, Umbrella review, Overview

## Abstract

Findings of high rates of complex trauma among justice-involved young people have engendered interest in developing trauma-informed youth justice systems. Although there have been several reviews of trauma-informed practice in youth justice settings, uncertainty remains about whether this approach can produce the outcomes expected of youth justice services. In this study we summarize findings from recent systematic reviews and meta-analyses to provide an overview of evidence relevant to implementing trauma-informed youth justice. We conducted an umbrella review of systematic reviews published between 2017 and 2023 that included group-based primary studies of trauma-informed interventions for justice-involved young people. Reviews were located via searches of PsycINFO, PubMedCentral, Embase, Criminal Justice Abstracts with Full Text, and ProQuest. Data extracted from each review included the number and type of primary studies reviewed, and outcomes related to trauma symptomatology, mental health and wellbeing, and justice system involvement. Nine systematic reviews met our inclusion criteria. Improvements in trauma symptoms, mental health and wellbeing, and justice system involvement were documented in each review. The strongest evidence related to the impact of trauma-focused interventions on posttraumatic stress disorder symptoms, but less evidence was available to demonstrate outcomes of organizational level and systemic components of trauma-informed practice. Each review highlighted the need to strengthen the methodological quality of primary studies. Trauma-informed practice should be seriously considered as part of any effort to implement evidence-based youth justice. This should extend beyond treatment of trauma symptomatology to incorporate a broader approach to trauma-informed practice that is organizationally embedded.

**Supplementary Information:**

The online version contains supplementary material available at 10.1007/s40653-024-00634-5.

Youth justice services around the western world have, in recent years, found themselves under increasing pressure to develop new and more effective ways of working. A series of reviews, inquiries, and investigations (e.g., the Carlile Inquiry, 2014; Clancey et al., 2020; Knox et al., 2013; RCPDCNT, 2017) have highlighted harmful and abusive practice, reigniting long-standing debates about the purpose of a youth justice system (e.g., Braithwaite & Mugford, 1994; Day, 2023) and identifying a need to develop practices that are evidence-informed and less punitive. It is in this context that the idea of ‘trauma-informed practice’ has emerged as a potentially promising way to develop new policies, programs, and interventions that can help to achieve better outcomes for children and young people while, at the same time, also ensuring the safety of the community. Indeed, a number of youth justice agencies around the world have now endorsed a trauma-informed approach (e.g., Jackson et al., 2023), even though it has been observed that terms such as ‘trauma-informed practice’ (TIP) and ‘trauma-informed care’ (TIC) often lack meaning. As a result, it is not always easy to determine if (or when) a trauma-informed approach is being implemented and, importantly, if it might be expected to lead to a reduction in clinically significant symptoms of trauma, to improvement in the wellbeing of justice-involved young people, and/or to a reduction in subsequent offending and other justice-related outcomes (Homes & Grandison, 2022). In this paper our aim is to provide an up-to-date summary of the current evidence relating to the outcomes of trauma-informed youth justice. This, we suggest, should be relevant information for policymakers when considering this approach, and serve to strengthen public confidence that trauma-informed models of service delivery will result in the desired outcomes of a justice agency.

## Trauma-Informed Youth Justice

Youth justice agencies typically strive to achieve different, but overlapping, aims. In Australia, for example, the federal government requires that youth justice services aim to promote community safety, to rehabilitate, and to reintegrate young people who offend, as well as to contribute to a reduction in youth re-offending (Report on Government Services, 2022). Thus, while the management of risk of reoffending is clearly a priority for all criminal justice agencies, there is also an expectation that the welfare of the child or young person should be protected and promoted. It is in relation to this philosophical change in how the community views its responsibilities towards justice-involved children and young people that interest in trauma-informed policy and practice has grown rapidly, with countries such as England and Wales now prioritizing child welfare over justice considerations (Haines & Case, 2015).

A trauma-informed approach is a universal approach that, at its core, is designed to do no further harm to those who have experienced traumatic life events (Liddle et al., 2016). The assumptions that underpin trauma-informed youth justice are, as a result, somewhat different from those that provide the foundation for the delivery of more traditional criminal justice services. As Griffin et al. (2012) have argued, trauma-informed work does not distinguish between ‘victim’ and ‘perpetrator’ in the same way that many western legal systems do; rather, ‘risk’ is conceptualized in terms of vulnerabilities that arise in response to childhood maltreatment and social and structural inequalities. Hence, the primary goal of service delivery is to provide an environment in which the impacts of maltreatment and adversity are acknowledged, processed, and resolved. A primary concern is to minimize exposure to potentially retraumatizing events. Thus, trauma-informed youth justice is not simply concerned with the provision of mental health services that address symptoms of trauma (this is usually referred to as ‘trauma-focused’ intervention and relates primarily to the provision of mental health treatment), but also to mitigate the risk of young people behaving, or being treated, in ways that re-traumatize or cause harm to others or to themselves (Buckingham, 2016). One way that youth justice agencies have sought to reduce re-traumatization, for example, is to provide a structured and safe environment (e.g., regular meals, bedtimes, school times, expectations of behavior) such that basic psychological and health needs are met (Skuse & Matthew, 2015).

The rationale for implementing trauma-informed practice is derived, in part, from research showing that many justice-involved children and young people have experienced – and may continue to experience – maltreatment and adversity. It is now well-established that across all youth justice systems, most children and young people will have a history of (often extensive) child protection system contact (see Lamers-Winkelman et al., 2012; Spinhoven et al., 2010), with experiences of victimization in childhood associated with both clinically significant symptoms of trauma (Malvaso et al., 2022) *and* offending (Berg & Schreck, 2022). In a recent review, for example, Yoder and Tunstall (2022) reported that as trauma exposure accumulates over time, so too do high-risk behaviors and contacts with the youth justice system (see also Layne et al., 2014).

There has been considerable interest in understanding the developmental pathways that result in offending. As de Ruiter et al. (2022) observed in their discussion of how maltreatment and trauma can increase risk, one possible mechanism is that the emotional numbing and feeling of detachment that often results from trauma leads to increases in callousness and disregard for victims. Another hypothesis is that exposure to traumatic stressors compromises secure attachment with primary caregivers, resulting in self-regulatory deficits that facilitate offending (Ford et al., 2012a, b). Alternatively, the degree to which maltreatment represents a ‘betrayal’ of trust may influence the way in which abuse-related information is processed and remembered and trigger antisocial behavior. Another consideration is the way in which systemic interventions mitigate or exacerbate trauma systems, such as the placement of children who have experienced maltreatment into out-of-home care. Placement in residential care facilities is known to exacerbate trauma symptoms and associated behavioral problems which, in turn, may lead to an increased risk of contact with the justice system (Malvaso & Delfabbro, 2015; Ryan et al., 2008).

The broad conclusion that can be drawn here is not only that trauma reactions are often a catalyst for involvement in the criminal justice system, but that they can also act to increase the risk of offending and re-offending (see Becker & Kerig, 2011; Craig et al., 2017; Ford et al., 2010). Put simply, the key presentations of trauma (e.g., impulsivity, risk-taking, and low self-control) represent important criminogenic needs (or ‘dynamic risk factors’; see Klepfisz et al., 2016), and should thus form important intervention targets for any service that aims to reduce re-offending (see Ford et al., 2007). It follows perhaps that a logical service response is not to ‘punish’ justice-involved young people and implement measures that deter them and others from offending, but to offer a more therapeutically aligned approach that helps children to feel safe and to gain control over intense reactions, destructive thoughts, and impulsive behaviors. The key point here, however, is that the aim of trauma-informed youth justice is, inevitably, to achieve multiple outcomes – and these include reducing trauma symptoms, promoting good mental health and wellbeing and reducing externalizing and offending behaviors.

Given that a trauma-informed youth justice will aim to influence different, and potentially conflicting, outcomes, there is a need for clarity about the processes, activities, and interventions that will best achieve these goals. In response to concerns that trauma-informed approaches to youth justice lack coherence, Branson and colleagues (2017) published a systematic review that identified core elements of service delivery as well as offering comprehensive recommendations for implementation and evaluation. Three domains of recommended practices were identified: (1) clinical services for youth involved in the justice system (screening and assessment, trauma-focused treatment, cultural competence); (2) agency context (young person and family engagement, workforce development, providing a safe environment, agency policies, procedures, and leadership); and (3) systems level (systems policy and procedures, cross-agency collaboration, quality assurance and evaluation). Branson et al. (2017) also recommended further research to establish which, if any, of these practices are effective in relation to both wellbeing and justice-related outcomes for children and young people. Since then, a series of systematic reviews and meta-analyses have been published that share a common aim of synthesizing the available evidence on trauma-informed approaches to youth justice. Each of these has a slightly different focus and, given the multiple components of a trauma-informed approach, the current evidentiary standing of trauma-informed programs, service delivery, and policy frameworks across youth justice is not easy to ascertain. This creates challenges for policy makers and practitioners who are seeking new and different ways of working. Accordingly, the purpose of this study is to curate current evidence to arrive at an overall assessment of this relatively new approach to youth justice service delivery.

## Method

An umbrella review methodology was used in this study to curate knowledge from systematic reviews and meta-analyses to determine the overall strength of evidence on a particular topic (Pollock et al., 2020). It is a structured approach that utilizes the Preferred Reporting Items for Systematic Reviews and Meta-analysis (PRISMA) statement (Page et al., 2021) and involves an assessment of the methodological quality of the reviews considered before summarizing the evidence. It also aids identification of differences in how reviews evaluate overlapping primary studies and interventions (Pollock et al., 2020). As all analyses reported in this study were based on previous published studies, no ethics approvals or participant consents were required.

### Search Strategy and Inclusion Criteria

To locate eligible studies, searches were conducted of the following databases: PsycINFO; PubMedCentral; Embase; Criminal Justice Abstracts with Full Text (EBSCOhost); ProQuest (Social Science Premium Collection); and CINCH Australian Criminology Database. We also searched the Cochrane Database of Systematic Reviews, the Campbell Collaboration, and PROSPERO International prospective register of systematic reviews. Given varying definitions and conceptualizations of trauma-informed practice, our search terms were intentionally broad to identify a range of relevant reviews, including those that while not labelled as ‘trauma-informed’ nonetheless included studies of trauma-informed or trauma-focused practices and interventions. To illustrate, the following search terms were entered into the PsycINFO data base, and adapted for other databases as necessary: “juvenile justice or juvenile delinquency/ or ((youth or juvenile or young or adolescen* or minor) adj3 (justice* or justice-involved or justice involved or court* or detention* or delinquen* or incarcerated or incarceration or correction* or offend* or custody)) and trauma-informed care.sh or exp treatment/ or exp treatment outcomes/ or (trauma* adj (informed or focused or responsive or oriented or specific)) and (systematic review or meta analy* or meta-analy*)”. Searches were conducted between October 2022 and April 2023.

To be included, a review had to have included at least one quantitative evaluation of a trauma-informed, or trauma-focused, group-based intervention aimed at improving outcomes for a justice-involved youth population (i.e., young people who were currently involved with the justice system, aged between 10 and 24 years, with at least some participants under the age of 18). The review had to be in the English language, peer-reviewed, and published in a five-year period following Branson et al.’s (2017) systematic review, with purely theoretical or policy-focused articles excluded. Reviews that involved only qualitative studies, individual case studies, or evaluations of the impact of a therapeutic environment in non-youth justice settings were also excluded. Given the scarcity of randomized studies in this field, reviews of randomized and non-randomized studies were included.

### Data Extraction and Management

**Fig. 1:**
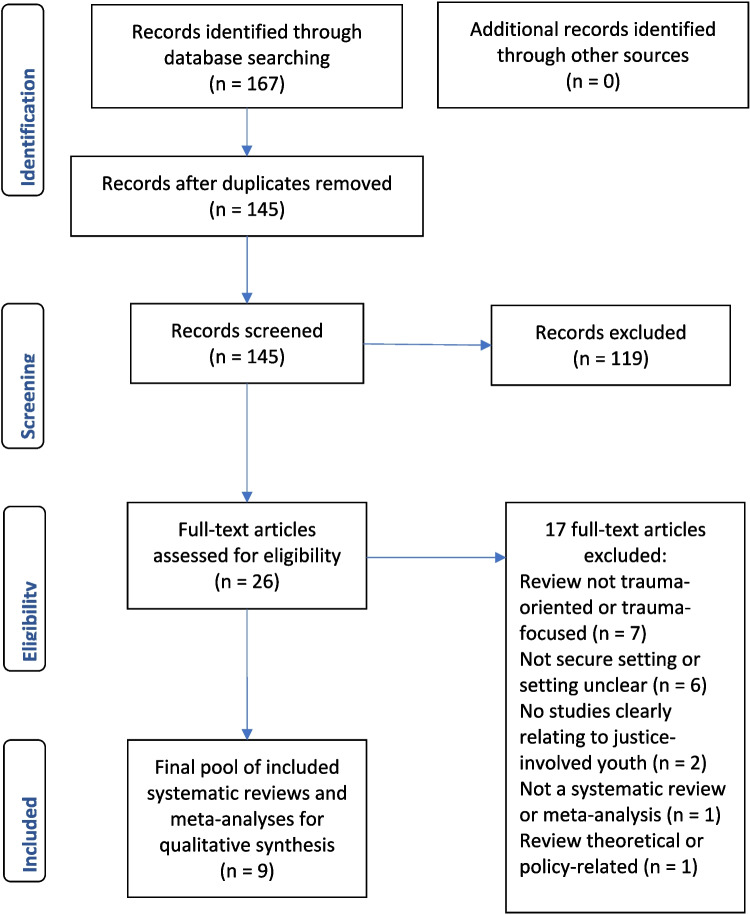
Flow chart of the study selection process

Review selection was undertaken by two authors using the Covidence systematic review software (Covidence, 2022). After removing duplicate records, the same two authors independently screened abstracts and read the full-text articles. Where there was disagreement, discussion ensued until full agreement was reached. The PRISMA flow chart of study selection can be found in Fig. 1.

Each review was independently coded, with reference to a template designed to describe key features and to report the main findings. The coding form examined several content areas: author and review descriptors (e.g., authors, year); sample descriptors (e.g., population, age range, intervention); design; conclusions; outcome (trauma symptoms, other mental health outcomes, and justice-related outcomes such as re-offending and institutional behavior); and a summary of key analyses/findings and review conclusions. PICO information (population, intervention, comparator, outcome) from the primary studies included in each review was also recorded, as recommended by Pollock et al., 2020. All authors consulted regularly during this process.

Two authors then assessed the reviews against the AMSTAR 2 quality indicators (Shea et al., 2017). Based on the original AMSTAR for quality assessment of systematic reviews of randomized studies (Shea et al., 2007), AMSTAR 2 incorporates additional items to address the greater susceptibility of non-randomized studies of interventions (NSRI) to bias, compared with randomized studies. Of the 16 items, three are specific to meta-analyses.

### Data Synthesis

A brief narrative description of each review was then produced, synthesizing the main findings relating to three principal outcome domains of trauma-informed youth justice (trauma symptomatology, mental health and wellbeing, and justice-related outcomes).

## Results

### Description of Included Reviews

Nine systematic reviews – three meta-analyses and six narrative reviews – met our inclusion criteria. Justice-involved children and young people were the target population in five of these reviews, while studies involving both justice-involved and non-justice-involved children and young people populations (such as at-risk youth or youth in psychiatric settings) were included in the remainder. Only findings related to group-based evaluations of interventions provided in justice-involved youth populations are reported in this study. A summary of the main characteristics of each review is presented in Table 1.

**Table 1:** Key Characteristics of included reviews

Review authors, year, type of review, country of origin	Number of included studies	Number of group-based studies with justice-involved youth (sample N)	Interventions	Intervention orientation and components	Outcome domain		
					Trauma	Mental health and wellbeing	Justice system related outcomes
Baetz et al. (2022) Systematic Review US	7	7 (655)	Cognitive processing therapy; SITCAP-ART; Think Trauma (staff training) + TGCT-A (youth treatment); TARGET (2 studies); MTFC + T; WRITE-ON	Trauma processing; sensory processing; CBT; trauma affect regulation education & skill development; TI environmental modifications; TI psychoeducation and training; expressive writing	PTSD symptoms	Co-occurring mental health symptoms and disorders	Juvenile justice-related outcomes (including behavioral problems, time spent in seclusion, post-release delinquent behavior and/or rearrest)
Eadeh et al. (2021) Meta-analytic review US	31	4 (304)	TARGET; Multiple group family intervention (2 studies); juvenile justice anger management for girls	Trauma affect regulation education and skill development; affect regulation and attachment; anger management.	-	Emotion regulation; maladaptive and adaptive affect regulation; positive and negative cognitive regulation	-
Gagnon et al. (2022) Systematic Review Finland/US	11	10 (1882)	Think Trauma (staff training) + STAIR (youth therapy); Addressing the Harm; dialectical behavior therapy; TF-CBT; motivational interviewing; gender-responsive programming (vs behavioral reinforcement); Sanctuary®; WRITE-ON; social problem-solving training; TGCT-A (youth therapy) + Think Trauma (staff training)	Trauma affect regulation education and skill development; restorative justice; CBT; trauma-informed organizational transformation; problem solving; trauma & grief processing; trauma-informed staff training	Trauma symptoms	Internalizing, depression; affect regulation; perceived safety	Violent incidents (youth); restraint and seclusion; recidivism
Givens et al. (2021) Systematic Review US	19	6 (448)	Meditation course; mindfulness training; TARGET; aerobic exercise training; TGCT-A (youth) + Think Trauma (staff); structured group therapy; coping course	Mindfulness; trauma affect regulation, psychoeducation and skill development; physical fitness training; CBT; coping & problem-solving; trauma & grief processing; staff training	-	Affect-regulation; externalizing behaviors; mood; life attitudes; self-esteem; social adjustment	-
Hodgkinson et al. (2021) Systematic Review UK	14	14 (5,469)	Goal setting + post-release care; re-entry services; cognitive training + post-release care; decompression treatment (2 related studies); enhanced thinking skills (vs reasoning & rehabilitation); Two intensive regimes for young offenders; TARGET; Teen Court; diversion program; Re-ART; computerized training in negative emotion recognition; cognitive intervention program; values-based therapeutic environment	A diverse range of psychological treatments aimed at developing psychological resilience in serious juvenile offenders. Components include CBT; positive behavioral reinforcement; intensive fitness training, vocational training, emotion recognition; problem-solving and goal setting; negative behavioral reinforcement	-	Psychological measures of resilience, emotion regulation, dynamic risk factors, self-esteem, sense of self, sense of coherence; psychopathy	Reoffending and related outcomes (frequency and severity of offending; time to reoffending; completion of community sentences)
Kumm et al. (2019) Meta-analytic review US	11	11 (821)	Cognitive processing therapy; dialectical behavior therapy; psychoeducational group intervention (2 unrelated studies); CBT; social problem-solving training; attributional retraining; TARGET; TGCT-A (youth) + Think Trauma (staff training); coping course; animal assisted therapy	Cognitive processing and cognitive behavioral therapy; psychoeducation; affect regulation (including distress tolerance); coping and problem-solving; animal therapy	PTSD	Anxiety, depression, internalizing behavior	-
Olaghere et al. (2021) Meta-analysis US	30	6 (393)	Cognitive processing therapy; TARGET (2 studies); MTFC; image rehearsal therapy; Sanctuary Model®	Cognitive processing, trauma affect regulation, psychoeducation and skill development; TI environmental modification; CBT and behavioral reinforcement; trauma processing; trauma-informed organizational transformation and psychoeducation	PTSD and related symptoms	Affective outcomes; hope	Delinquent behavior
Purtle (2020) Systematic Review US	23	1 (30)	Sanctuary Model	Trauma-informed organizational transformation	-	Physical and perceived safety of youth and staff	-
Rhoden et al. (2019) Systematic Review US	14	12 (995)	Cognitive processing therapy; TF-CBT training (staff training) + TFCBT (youth); TARGET (3 studies); MASTR; SPARCS; prolonged exposure; image rehearsal therapy; structured group therapy; psychoeducational group therapy; SITCAP-ART; EMDR; MTFC + T	Cognitive processing; CBT; exposure; trauma processing; sensory processing; eye movement desensitization; trauma affect regulation, psychoeducation and skill development; trauma-informed environmental modification; goal setting, behavioral reinforcement	PTSD symptoms	-	-

*CBT* cognitive-behavioral therapy, *EMDR* eye movement desensitization and reprocessing, *MASTR* Motivation-Adaptive Skills-Trauma Resolution, *MTFC* Multidimensional Treatment Foster Care, *MTFC* + *T* Multidimensional Treatment Foster Care plus Trauma, *RE-ART* Responsive Aggression Regulation Therapy (Outpatient), *SITCAP-ART* Structured Sensory Intervention for Traumatized Children, Adolescents and Parents – Adjudicated and at Risk Youth, *SPARCS* Structured Psychotherapy for Adolescents Responding to Chronic Stress, *TARGET* Trauma Affect Regulation: Guide for Education and Therapy, *TF-CBT* trauma-focused cognitive behavioral therapy, *TGCT-A* Trauma and Grief Component Therapy-Adolescent, *TI* trauma-informed, *WRITE-ON* Writing and Reflecting on Identity To Empower Ourselves as Narrators, *PTSD* posttraumatic stress disorder

The nine reviews encompassed a total of 47 group-based juvenile justice-related primary studies (1 to 14 per review), with 15 of the primary studies included in more than one review (see Table S1 in the supplementary material). Accounting for duplicates (n = 2245), there were 8615 participants (n = 30 to n = 5469 justice-involved young people per review). Most participants were male, and ages ranged from 11 to 24 years. All nine reviews included one or more controlled studies (i.e., randomized, or quasi-experimental designs with comparison group). Seven of the reviews also included group studies without a comparison group (e.g., single-group, pretest–posttest), and two included individual case studies. The sample size of primary studies included in each review ranged from n = 9 to n = 3068.

In total, 41 named interventions targeting outcomes relevant to justice-involved youth were evaluated. Among these were 29 group-based manualized programs, of which the majority were psychological treatments, either based on cognitive-behavioral therapy (CBT) or incorporating elements of it. Among the trauma-specific elements of treatment were psychoeducation about trauma and its effects on psychosocial development and emotion regulation; skill development in self-awareness, emotion regulation; mindfulness and meditation training; processing trauma-related memories (e.g., through trauma narratives); sensory processing; and dealing with future trauma. One intervention focused on organizational transformation. Most of the psychological interventions also included therapist training prior to delivering treatment. In only a few cases, however, was a specific staff training program named as a component of the intervention.

#### Methodological Quality

The results of the AMSTAR 2 assessment of methodological quality are reported in Table 2. Generally, studies wholly or partially met four of the criteria: specifying PICO characteristics in their inclusion criteria, using a comprehensive literature search strategy, reporting studies in sufficient detail, and reporting funding and/or conflicts of interest. However, no study fully met all criteria, as none provided a list of excluded studies with reasons for exclusion or reported funding information for all included primary studies. The reviews differed in terms of whether they had developed an a priori review protocol, provided an explanation for selection of study designs, performed study selection and data extraction in duplicate, or formally assessed sources of individual study bias. Several reviews attained only a ‘partial yes’ for adequate primary study description because design features, such as the number of groups or the type of control condition were unclear. Studies also varied in the completeness with which they reported relevant aspects of sample characteristics such as racial composition, and mental health diagnosis. All but one of the reviews had undertaken some form of quality assessment. Five (Gagnon et al., 2022; Givens et al., 2021; Hodgkinson et al., 2021; Kumm et al., 2019; Rhoden et al., 2019) evaluated primary studies against existing assessment tools, while two (Baetz et al., 2022; Rhoden et al., 2019) assessed sources of bias using the Cochrane Risk of Bias tool. One meta-analysis assessed the reliability and validity of included outcome measures (Eadeh et al., 2021), while another formally assessed the risk of publication bias (Olaghere et al., 2021).

**Table 2:** AMSTAR 2 Ratings of included reviews

	**AMSTAR 2 Rating Domains**															
Review First Author	**1**	**2**	**3**	**4**	**5**	**6**	**7**	**8**	**9**	**10**	**11**	**12**	**13**	**14**	**15**	**16**
Baetz et al. (2022)	**Y**	**Y**	**Y**	**Y**	**Y**	**Y**	**N**	**Y**	**Y**	**N**	**NMA**	**NMA**	**PY**	**Y**	**NMA**	**Y**
Eadeh et al. (2021)	**Y**	**PY**	**N**	**PY**	**NR**	**NR**	**N**	**PY**	**N**	**N**	**Y**	**N**	**PY**	**Y**	**N**	**Y**
Gagnon et al. (2022)	**Y**	**PY**	**N**	**PY**	**NR**	**Y**	**N**	**PY**	**N**	**N**	**NMA**	**NMA**	**N**	**N**	**NMA**	**Y**
Givens et al. (2021)	**Y**	**Y**	**PY**	**PY**	**NR**	**NR**	**N**	**PY**	**PY**	**N**	**NMA**	**NMA**	**N**	**Y**	**NMA**	**Y**
Hodgkinson et al. (2021)	**Y**	**N**	**PY**	**Y**	**Y**	**NR**	**N**	**PY**	**PY**	**N**	**NMA**	**NMA**	**N**	**Y**	**N**	**Y**
Kumm et al. (2019)	**Y**	**PY**	**Y**	**Y**	**Y**	**Y**	**N**	**PY**	**N**	**N**	**PY**	**N**	**PY**	**N**	**N**	**N**
Olaghere et al. (2021)	**Y**	**PY**	**PY**	**Y**	**NR**	**NR**	**N**	**PY**	**NR**	**N**	**Y**	**Y**	**Y**	**Y**	**Y**	**Y**
Purtle (2020)	**N**	**N**	**N**	**PY**	**N**	**N**	**N**	**PY**	**N**	**N**	**NMA**	**NMA**	**Y**	**N**	**NMA**	**y**
Rhoden et al. (2019)	**Y**	**N**	**N**	**Y**	**N**	**NR**	**N**	**Y**	**Y**	**N**	**NMA**	**NMA**	**Y**	**Y**	**NMA**	**Y**

Domains: 1 = Did the research questions and inclusion criteria for the review include the components of PICO?; 2 = Did the report of the review contain an explicit statement that the review methods were established prior to the conduct of the review and did the report justify any significant deviations from the protocol?; 3 = Did the review authors explain their selection of the study designs for inclusion in the review? 4 = Did the review authors use a comprehensive literature search strategy?; 5 = Did the review authors perform study selection in duplicate?; 6 = Did the review authors perform data extraction in duplicate?; 7 = Did the review authors provide a list of excluded studies and justify the exclusions?; 8 = Did the review authors describe the included studies in adequate detail?; 9 = Did the review authors use a satisfactory technique for assessing the risk of bias (RoB) in individual studies that were included in the review?; 10 = Did the review authors report on the sources of funding for the studies included in the review?; 11 = If meta-analysis was performed did the review authors use appropriate methods for statistical combination of results?; 12 = If meta-analysis was performed, did the review authors assess the potential impact of RoB in individual studies on the results of the meta-analysis or other evidence synthesis?; 13 = Did the review authors account for RoB in individual studies when interpreting/discussing the results of the review?; 14 = Did the review authors provide a satisfactory explanation for, and discussion of, any heterogeneity observed in the results of the review?; 15 = If they performed quantitative synthesis did the review authors carry out an adequate investigation of publication bias (small study bias) and discuss its likely impact on the results of the review?; 16 = Did the review authors report any potential sources of conflict of interest, including any funding they received for conducting the review?

Answers: *Y* Yes, *PY* Partial Yes, *N* No, *NMA* No meta-analysis conducted, *NR* Not reported

#### Narrative Description of Main Findings (in alphabetical order of review)

Baetz et al. (2022) systematically reviewed seven controlled studies published between 2002 and 2017 to examine the impact of manualized, trauma-specific treatments on the reduction of post-traumatic stress disorder (PTSD) symptoms, co-occurring mental health symptoms, and justice-related outcomes in justice-involved young people. Treatment effect sizes were calculated for PTSD. Four studies were reported as showing a significant decrease in PTSD symptoms following treatment when compared with a control group. Regarding improvement in mental health symptomatology, both cognitive processing (Ahrens & Rexford, 2002) and TARGET (Marrow et al., 2012) were associated with reductions in depressive symptoms, but in the case of Marrow et al. it was noted that the magnitude of the difference in outcome between the TARGET and control groups may have reflected an increase in depression in the control group. The two studies that examined recidivism produced conflicting results, with Multidimensional Treatment Foster Care plus Trauma (MTFC + T; Smith et al., 2012) showing reductions in delinquency and recidivism in adolescent girls, and TARGET (Ford & Hawke, 2012) showing no differences in rearrests between the treatment and control groups. Baetz et al. suggested that the differing results may have reflected differences in how recidivism was operationalized, as well as greater follow-up care in the Smith et al. study.

Overall, Baetz et al.’s (2022) review concluded that evidence for the effectiveness of trauma specific treatments with young people in justice settings was encouraging (p. 650) However, the review also noted a lack of methodologically sound studies, along with several challenging aspects of implementation. These included integrating new treatments into existing practices; engaging stakeholders; monitoring treatment fidelity; and ensuring that daily care staff in secure settings are adequately trained and involved in program delivery. Potential sources of study bias that were identified included a high risk of incomplete outcome reporting and/or selective reporting. The authors advised that the results of the respective studies should be interpreted with caution.

Eadeh et al. (2021) conducted a meta-analytic review of evidence from 41 studies of the effects of emotion regulation interventions in adolescents with a wide range of presenting disorders, including trauma. The underlying premise was that a lack of adaptive emotion regulation strategies and a reliance on maladaptive strategies are risk factors for the development of internalizing and externalizing disorders that are linked to poor adolescent outcomes. Gross’s (1998, 2015) model (identifying emotional responses to situations, and selecting and implementing strategies to manage them) was used to conceptualize emotion regulation.

Four of the studies reviewed (two randomized control trials and two single-group studies) focused on incarcerated and delinquent adolescents. Where data were available, Hedges’ *g* was used to calculate intervention effects (Keiley, 2007, did not provide means and standard deviations for the outcomes, so the original results were reported). Three studies were reported as showing significant improvements following the emotion regulation interventions. Keiley (2007) reported significant decreases in incarcerated adolescents’ self- and maternal caregiver reports of maladaptive emotion regulation following participation in a multiple-group family intervention (MGFI) program (fathers’ reports did not improve). Keiley and colleagues (2015) found similar improvements following implementation of MGFI in a sample of male adolescent sexual offenders. Ford et al. (2012a, b) found that TARGET was associated with a significant reduction compared with treatment as usual in negative emotion regulation in girls placed in a juvenile justice facility. A fourth study, evaluating a juvenile justice anger management intervention for girls, was reported as not delivering improvements in emotion regulation compared with treatment as usual (Riggs Romaine et al., 2018).

Results from the three studies with calculated effect sizes were included in the pooled effect size analysis across all studies. Results showed significant positive treatment effects for both maladaptive (negative) and adaptive (positive) emotion regulation strategies, albeit that there was considerable heterogeneity and effects were small. The authors determined that studies involving clinical samples demonstrated larger treatment effects than those involving non-clinical samples, and this was the case for both single-group and controlled studies. Accordingly, they recommended that future research should include more ‘methodologically rigorous comparison groups’ (p. 701). The authors also recommended increased inclusion of measures that detect changes in the use of positive emotion regulation and coping strategies.

Gagnon et al.’s (2022) systematic review considered evaluations of mental health interventions for incarcerated young people. Only primary studies published since the release of *Guiding Principles for Providing High-Quality Education in Juvenile Justice Secure Care Settings* (U.S. Departments of Justice and Education, 2014) were included. Eleven studies met the authors’ inclusion criteria, of which ten were quantitative, group-based studies. Although the included interventions varied in emphasis, most incorporated elements of CBT. An exception was the Sanctuary program, which aims to promote a safe and therapeutic environment through organizational transformation, and staff education and training (Elwyn et al., 2015). Two of the CBT-based interventions also included a component of staff trauma training (see Table S1 in the supplementary material for this review), but staff outcomes were not reported. Outcomes studied included mental health symptoms (posttraumatic stress disorder, depression), justice-related outcomes (institutional violence, recidivism, institutional safety), and personal growth (resilience, changes in dynamic risk and protective factors). Positive main effects for at least one of the studied outcomes in each study were reported for seven of the ten interventions. For two of the remaining three, moderator analyses showed that interventions were effective for some participants, but not others. Specifically, a motivational interviewing intervention for incarcerated girls was associated with reduced substance-related predatory aggression in girls with lower, but not higher, levels of depression, while social problem-solving training was associated with reduced depressive symptoms in males with higher intelligence but appeared to exacerbate symptoms in those with less high intelligence. One quasi-experimental study of the effect of a restorative justice program on criminogenic risk and protective factors was reported as indicating no superior effect of assignment to the treatment program over assignment to a control condition in which participants watched a series of short victim impact videos. However, treatment completers showed improved skills in impulsivity control and in dealing with their own and others’ feelings. Overall, treatment effects appeared to be stronger for males than females. The study’s high attrition rate was noted (Gagnon et al.).

Despite noting multiple methodological shortcomings across the reviewed studies, Gagnon et al. (2022) recommended four treatments for use in youth justice facilities — trauma-focused CBT, motivational interviewing, trauma and grief component therapy, and dialectical behavior therapy — as having a prior evidence base in non-youth justice populations, as well as demonstrating a positive outcome in the reviewed studies. However, as indicated above, motivational interviewing did not reduce predatory aggression in all participants; rather, it only reduced aggression among those girls with low levels of depressive symptomatology. This finding – as well as the results of other moderator analyses noted above—underscores the need to consider individual differences when selecting treatments for justice-involved youth. Sanctuary, dialectical behavior therapy, the Think Trauma staff training program, and STAIR were also cautiously recommended for further study and evaluation, while a gender-responsive program and the restorative justice program described above were not (the latter based on the assignment to treatment results reported above). The authors noted the need to establish confidence in treatment integrity and the need for stronger research methodology.

Givens et al. (2021) conducted a systematic review of original studies carried out in the United States and determined to be valid using National Institutes of Health study quality assessment tools. Of 19 identified original studies, six (published between 1988 and 2018) were conducted in youth correctional facilities. Interventions included CBT and its variations, as well as intensive mindfulness meditation, physical exercise training, TARGET, and coping skill training. All the cognitive-behavioral interventions resulted in significant reductions in posttraumatic stress disorder, while TARGET and physical exercise training were associated with improved mood, and coping training was associated with improved self-esteem, reduced externalizing, and reduced death-related life attitudes. The effect of the intensive mindfulness meditation intervention (a seven-hour meditation retreat) was reported as not being significant. However, in the latter study, the control group, as well as the intervention group, was assigned to a ten-session mindfulness meditation curriculum, following which the combined results of both groups showed an overall improvement in self-regulation. The review authors concluded that the variety of interventions, outcomes, study settings, and implementation procedures made efforts to synthesize the evidence difficult.

Hodgkinson et al. (2021) systematically reviewed 14 studies, published between 2001 and 2018, which had documented reductions in recidivism among repeat youth offenders following implementation of psychological resilience interventions. This review includes studies from Europe as well as the US. The review authors emphasized the role of childhood trauma in impeding cognitive and emotional development in young people, leading to high levels of negative emotions and reactivity in those who subsequently become involved in the justice system. They further argue that psychological resilience may act as a protective factor against the risk of offending among adolescents who have experienced childhood trauma, while interventions that are explicitly designed to promote psychological resilience among youth who have already offended could be effective in reducing their risk of re-offending.

A wide range of treatments (see Table 1) was reviewed, including one explicitly trauma-informed program (TARGET; Ford & Hawke, 2012). Among the mechanisms considered responsible for positive treatment effects were an improved sense of coherence and an increased capacity to recognize emotions in others, since post-intervention improvements in these aspects of resilience were associated with observed reductions in reoffending. An increased sense of empowerment and improved decision-making in young people were also suggested as possible explanations.

Kumm et al. (2019) conducted a meta-analytic review of mental health interventions in juvenile justice facilities for young people with internalizing disorders. Eleven studies published between 1993 and 2017 were identified, of which seven included a control group (including four randomized control trials), and four employed a single-group, pretest–posttest design. In addition to CBT, dialectical behavior therapy, and TARGET, the interventions reviewed included animal assisted therapy and attributional retraining. Effect sizes with confidence intervals for individual studies were calculated, and meta-analyses of pooled effects were carried out. No interventions in studies with a control condition were associated with significant treatment effects on any outcomes. However, meta-analyses of single-group studies showed significant positive effects of treatment on internalizing symptoms, trauma, and depression, although not on anxiety. Review authors highlighted several methodological limitations associated with single-group studies (such as confounding the effect of time with the effect of treatment on the outcomes) and recommended that results be interpreted with caution. They also highlighted the need for more rigorous research and monitoring of treatment fidelity and recommended exploration of innovative study designs and interventions that cater better for short-stay residents.

Olaghere et al.’s (2021) meta-analysis and accompanying technical report (Wilson et al., 2018) reported on outcomes following the delivery of trauma-informed interventions in controlled studies of young people identified as at risk of justice involvement (23 studies) or justice-involved (six studies). The results of the six juvenile justice-related studies were reported separately from those for at-risk young people. Interventions examined were TARGET (Ford et al., 2012a, b; Marrow et al., 2012), cognitive processing therapy (Ahrens & Rexford, 2002), Multidimensional Treatment Foster Care (Chamberlain et al., 2007), Image Rehearsal Therapy (Krakow et al., 2001), and a version of Sanctuary that included programming for young people (Rivard et al., 2003) as well as techniques for therapeutic organizational transformation. For each study, standardized mean difference effect sizes for the outcomes were calculated (see Wilson et al., 2018, for full details) and were subsequently combined in meta-analyses. Confidence intervals (95%) and heterogeneity statistics were also reported.

Outcome domains included PTSD and trauma symptoms, affect (mental health), justice (delinquency, restraint), and hope. Three of four studies examining PTSD-related outcomes were reported as having a near null average effect size, with the remaining study (Krakow et al., 2001) observing a very large effect (*g* > 1.00) in a small sample. The meta-analytic means for PTSD outcomes and affect were reported as positive and small. One study that examined justice-related outcomes (Chamberlain et al., 2007) found that trauma treatment was associated with fewer criminal referrals and days locked up in an institution (Wilson et al., 2018); however, when these measures were combined with self-reported measures of delinquency in the same study, the resulting effect of trauma treatment was reported as essentially null (p. 1267). The other study that examined justice outcomes (Ford et al., 2012a, b) indicated slightly negative effects of TARGET on Child Behavior Checklist measures of delinquency, aggression, and externalizing behaviors.

Overall, based on a small number of studies, Olaghere et al. (2021) concluded that evidence that trauma-informed programs improve outcomes for justice-involved youth is modest and inconclusive, although nevertheless encouraging (p. 1267). Two interventions (cognitive processing and image rehearsal therapy) were named as indicating positive effects across a range of outcomes, but as previously noted, the relevant studies had small sample sizes (Ahrens & Rexford, 2002; Krakow et al., 2001). While the evidence for at-risk youth was stronger, the collection of reviewed studies overall was reported to be at high risk of publication bias favoring studies with significant results (see Wilson et al., 2018 for analyses). Olaghere et al. (2021) recommended that high-quality randomized experimental studies be conducted in future to isolate the effects of specific aspects of trauma-informed interventions on outcomes for young people who are justice-involved or at risk of justice involvement.

Purtle (2020) conducted a systematic review of trauma-informed organizational interventions with a staff training component. Twenty-four studies, published since 2000, were reviewed**.** One study, an evaluation of the Sanctuary Model, was conducted in a juvenile justice facility for girls (Elwyn et al., 2015), with the remainder being conducted in child welfare, health, residential care, and educational settings. The review concluded that Sanctuary resulted in improvements to physical and perceived safety for both staff and young people, but that its multifaceted approach made it difficult to isolate the effect of staff training.

Purtle (2020) concluded that the pool of reviewed studies offered sufficient evidence to indicate that participation in trauma-informed staff training resulted in improvements across a range of settings in staff knowledge, attitudes, and behaviors regarding trauma-informed practices. However, the duration of these benefits for staff was considered unclear, as was the extent to which they would result in improvements for clients. While noting various methodological shortcomings of reviewed studies (e.g., the predominance of single group studies, failure to use validated measures, limited or non-existent follow-up, and failure to disentangle the effects of multiple interventions), this review presents comprehensive recommendations to guide future research on trauma-informed staff training and related organizational interventions, while also summarizing the implications for practice and policy.

Rhoden et al.’s (2019) systematic review examined peer-reviewed studies conducted in the United States of trauma-specific interventions among justice-involved young people to the age of 21 with “reported traumatic exposure and/or PTSD symptoms based on a DSM diagnosis or a standardized measure” (p. 894). Sixteen studies, published between 2001 and 2016, were identified that met the inclusion criteria. Of these, twelve (ten controlled studies and two with a single-group design) were group-based with quantitative analysis of outcomes. Cognitive-behavioral principles were applied in most of the interventions studied, with treatment protocols typically including an educational component, skill-building, and self-regulation strategies. One study investigated eye movement desensitization and reprocessing therapy (EMDR).

Treatment effect sizes (Cohen’s *d*) were calculated based on reanalysis of individual study data. Medium to large effects of treatment on PTSD and/or other trauma symptoms were reported for nine studies, while a small effect was reported for a tenth. For proxy measures of externalizing behavior (e.g., time spent in seclusion, delinquency), small to large treatment effects were found in three studies. The studies of EMDR and trauma-focused CBT were considered to present the strongest evidence of treatment effectiveness based on the review authors’ assessments of their comparative methodological rigor. However, Rhoden et al. (2019) concluded that there was insufficient high-quality evidence to indicate that trauma interventions reduce trauma symptoms *and* externalizing behavioral problems despite their co-occurrence.

## Discussion

The main findings from this umbrella review are that the provision of trauma-focused interventions is associated with a decrease in trauma symptoms in justice-involved populations (with cognitive-behavioral approaches receiving the strongest empirical support), as well as with improvement in co-occurring mental health problems. There is also evidence of a positive impact on different metrics of re-offending and institutional misconduct. Based on our reading of the available evidence, this offers a sufficiently strong rationale to provide trauma-informed interventions to the broader youth justice population. This advice, while not particularly surprising (given that most of the review authors seem to agree), is nonetheless important in a context in which debates are ongoing about the need to establish safety and stabilization before trauma treatment can be provided. Concerns are, for example, often expressed that non-specialist treatment for trauma-related presentations can cause harm, with trauma treatment services sometimes only made available through external service providers, (such as child and adolescent mental health services) to those who meet the diagnostic criteria for posttraumatic stress disorder. The evidence reported in this study suggests that there are effective programs and interventions that can be embedded within a youth justice service that do not focus exclusively on treating presentations of posttraumatic stress. This is relevant to service planning as this type of treatment is necessarily focused on how exposure to a specific trauma, as a past event, leads to a sense of current threat. From a trauma-informed perspective, however, it is continuous actual or threatened traumatic events that are more significant, where an ongoing sense of threat becomes adaptive and necessary for survival (Rosenberg et al., 2008). Thus, efforts to implement trauma-informed youth justice should extend clinical models of service delivery to support resilience and recovery from the wide range of adverse childhood experiences that justice-involved young people have typically experienced. Effectiveness is, of course, not the only way in which the success of an intervention can be determined, with factors such as acceptability, adoption, appropriateness, cost, feasibility, fidelity, access to service, and sustainability (Brownson et al., 2012) as well as cultural and context-specific adaptations (Yim et al., 2024) also important. The body of research curated across these nine reviews also shows that such approaches are indeed feasible for delivery to youth justice populations across a range of different settings.

### Limitations

An important caveat to any recommendation to implement trauma-informed youth justice, however, is that nearly all the review authors specifically comment on the importance of addressing a range of implementation and integrity challenges if a stronger evidence base is to develop. They also all note the limitations in the methodological quality of the pool of primary studies that were included in their reviews. For example, several reviews noted that single-group studies tended to show greater treatment effectiveness (larger effect sizes) than studies with a control group, reflecting possible confounding of treatment with non-treatment effects (Eadeh et al., 2021; Kumm et al., 2019; Olaghere et al., 2021). In addition, the finding of comparatively large treatment effects in primary studies with small samples may indicate a publication bias through which, it is suggested, small studies are (even) more likely than studies with larger samples to be published because of significant findings (Hong et al., 2020). The need for more complete reporting of study information to overcome the possibility of selective reporting of results is noted (Baetz et al., 2022; Olaghere et al., 2021).

While calling for greater methodological rigor, the review authors also acknowledge the challenges that arise when conducting evaluation research in youth justice settings. Contextual factors that potentially affect the quality of evidence available include the high turnover of children and young people in youth justice settings, and the resulting impact on study attrition and sample sizes. High staff turnover rates also mean that new staff must be trained, and trust with children and young people re-established. Staff turnover also contributes to poor treatment fidelity, as do the lack of program supervision, lack of leadership, and poor organizational support and culture.

An additional set of limitations relates to specific aspects of the reviews themselves. One is that in a few cases, differences in methods used to synthesize findings produced different evaluations of the same intervention. Two examples of this are the impact of WRITE-ON (Greenbaum & Javdani, 2017) on shame, and the impact of TARGET (Marrow et al., 2012) on PTSD. While such differences may be relatively minor, they highlight the need for the reader to exercise care when drawing conclusions. Importantly, there were also differences between reviews in how well they captured aspects of sample diversity such as race and ethnicity in their descriptions of primary study characteristics. This made it difficult to gauge how closely the study samples resembled the wider population of justice-involved young people in their respective countries or regions of origin, particularly in the United States, where most of the primary studies were conducted. An additional limitation was that only three of the reviews included studies from other countries or regions, such as Canada, the UK, or Europe. There is clearly a need to extend research into trauma-informed youth justice to other geographical and cultural regions. And we would also note that, even among western countries, there are differences in judicial systems and the composition of justice-involved youth populations. For example, in former British colonies such as Canada, Australia, and New Zealand, it is First Nations populations who are especially overrepresented in the youth justice systems, reflecting the need to develop service responses that also acknowledge a range of social and economic disadvantages, such as poverty, social and health inequalities, systemic racism and discrimination, and intergenerational trauma.

Finally, this umbrella review was limited in scope by excluding non-peer-reviewed documents and other grey literature. And, although not by design, a major focus of the available research was on the outcomes of trauma-focused treatments and programs, which constitute only one aspect of trauma-informed juvenile justice practice (Branson et al., 2017).

### Next Steps for Advancing Trauma-Informed Youth Justice

There is an ongoing need to better understand the outcomes of the broad range of practices that constitute trauma-informed practice. Some kind of trauma awareness training for staff is, for example, considered a minimum requirement (Branson et al., 2017), and there is a consensus that to deliver this requires adequate resourcing and sustained organizational and system-level support, including leadership. There are elements of training in many of the primary studies that were included in the reviews that met the inclusion criteria in our study, but it was not easy to ascertain their impact on outcomes. The Elwyn et al. (2015) study, for example, describes an organizationally framed intervention (Sanctuary) and is featured in two of the reviews (Gagnon et al., 2022; Purtle, 2020), while the Marrow et al. (2012) evaluation of TARGET features environmental modifications as well as training (i.e., it goes beyond treatment). It is reasonable to conclude that the evaluation of organizational, systemwide interventions in youth justice settings is still less well developed than that of trauma-focused treatments. Without considering the impact of activity at every level of the organization (SAMHSA, 2014), the evaluation of specific programs is always going to be unsatisfactory. And so, we need to think about utilizing stronger research designs that can account for the ways in which individual level, group level, and organizational level components of trauma-informed practice interact and combine to produce the range of outcomes that youth justice services are expected to deliver.

An obvious next step will also be to better understand the experiences and views of young people in the youth justice system. The SAMHSA (2014) trauma-informed principles were reportedly developed with expert and public input with 2000 respondents and 20,000 comments/endorsements (Heris et al., 2022). This was a process specifically designed to ensure that the resulting principles reflected the voices of trauma survivors, and it thus becomes important to listen to what justice-involved young people have to say about the services they receive (see Day et al., 2023). The task then is to find new ways to triangulate outcome data with the experiences and insights of justice-involved young people.

Finally, there appears to be a need to further develop systems of audit, accountability, and accreditation to ensure that trauma-informed youth justice is being implemented in a way that can be expected to result in the specified desired or agreed upon outcomes for young people and the wider community. It has, for example, been observed that the connection between activity and outcomes is often implicit or absent in accounts of trauma-informed practice (Bazemore, 2006) and there may well be differences in the extent to which different parts of any justice system align with a trauma-informed philosophy. External youth justice stakeholders and mental health providers who work with trauma may, for example, focus more on symptom reduction and/or on promoting health and wellbeing, while the principal concern of youth justice staff will typically be to reduce risk and future justice system involvement. Our conclusion then is that, although there is much more work to do to achieve trauma-informed youth justice, it is a promising line of inquiry that is supported by evidence and can lead to better outcomes for both justice-involved young people and for communities. At a time in history when the need for new and innovative approaches has been identified, it remains a promising alternative to more punitive approaches.

## Electronic Supplementary Material

Below is the link to the electronic supplementary material.

Supplementary Material 1 (DOCX 39.0 KB)
