# Supplementary material for: The Outcomes of Trauma-Informed Practice in Youth Justice: An Umbrella Review
Source: J Child Adolesc Trauma. 2024 Apr 22;17(3):939–55. doi: 10.1007/s40653-024-00634-5 (PMC11413302; doi:10.1007/s40653-024-00634-5)
Supplement: Supplementary file 1 — Supplementary Material 1 (DOCX 39.0 KB) [file 40653_2024_634_MOESM1_ESM.docx]

The Outcomes of Trauma-Informed Practice in Youth Justice: An Umbrella Review. Supplementary Information

Table S1. Group-based primary studies featured in included systematic reviews and meta-analyses.

| List of primary studies | | | Included systematic reviews and meta-analyses (first author) | | | | | | | | |
| --- | --- | --- | --- | --- | --- | --- | --- | --- | --- | --- | --- |
| Citation | Intervention | Number of times reviewed | Baetz  (2022) | Eadeh (2021) | Gagnon (2022) | Givens (2021) | Hodgkinson (2021 | Kumm (2019) | Olaghere (2021) | Purtle (2020) | Rhoden (2019) |
| Ahrens & Rexford (2002) | Cognitive Processing Therapy (CPT) | 4 | ^a^ x | -- | -- | -- | -- | x | x | -- | x |
| Baetz et al. (2021) | Think Trauma (staff training) + STAIR (youth program) | 1 | -- | -- | x | -- | -- | -- | -- | -- | -- |
| Baglivio & Jackowski (2015) | Addressing the Harm (Restorative Justice) | 1 | -- | -- | x | -- | -- | -- | -- | -- | -- |
| Bahr et al. (2016) | Goal setting, phone-calls, aftercare | 1 | -- | -- | -- | -- | x | -- | -- | -- | -- |
| Banks et al. (2015) | Dialectical Behavior Therapy (DBT) | 2 | -- | -- | x | -- | -- | x | -- | -- | -- |
| Barnert et al. (2014) | Intensive meditation program (retreat) | 1 | -- | -- | -- | x | -- | -- | -- | -- | -- |
| Bouffard et al. (2008) | Re-entry services | 1 | -- | -- | -- | -- | x | -- | -- | -- | -- |
| Braboy (2014) | Psychoeducational Group Intervention | 1 | -- | -- | -- | -- | -- | x | -- | -- | -- |
| Burraston et al. (2014) | Cognitive training & phone follow-up | 1 | -- | -- | -- | -- | x | -- | -- | -- | -- |
| Caldwell & Rybroek (2001) | Decompression Treatment | 1 | -- | -- | -- | -- | x | -- | -- | -- | -- |
| Caldwell et al. (2006) | Decompression Treatment | 1 | -- | -- | -- | -- | x | -- | -- | -- | -- |
| Calleja (2020) | TF-CBT | 1 | -- | -- | x | -- | -- | -- | -- | -- | -- |
| Cann et al. (2005) | Enhanced Thinking Skills | 1 | -- | -- | -- | -- | 1 | -- | -- | -- | -- |
| Chamberlain et al. (2007) | MTFC | 1 | -- | -- | -- | -- | -- | -- | x | -- | -- |
| Clair-Michaud et al. (2016) | Motivational Interviewing | 1 | -- | -- | x | -- | -- | -- | -- | -- | -- |
| Cohen et al. (2016) | TF-CBT (staff training); TFCBT (youth treatment) | 1 | -- | -- | -- | -- | -- | -- | -- | -- | x |
| Day, A. et al. (1993) | CBT brief therapy | 1 | -- | -- | -- | -- | -- | x | -- | -- | -- |
| Day, J. et al. (2015) | Gender-responsive programming | 1 | -- | -- | x | -- | -- | -- | -- | -- | -- |
| Elwyn et al. (2015) | Sanctuary | 2 | -- | -- | x | -- | -- | -- | -- | x | -- |
| Farrington et al. (2002) | Two Intensive Regimes for Young Offenders | 1 | -- | -- | -- | -- | x | -- | -- | -- | -- |
| Ford & Hawke (2012) | TARGET | 3 | x | -- | -- | -- | x | -- | -- | -- | x |
| Ford, Steinberg, et al. (2012) | TARGET | 3 | -- | x | -- | -- | -- | -- | x | -- | x |
| Forgays et al. (2005) | Teen Court | 1 | -- | -- | -- | -- | x | -- | -- | -- | -- |
| Greenbaum & Javdani (2017) | WRITE-ON | 2 | x | -- | x | -- | -- | -- | -- | -- | -- |
| Habib et al. (2001) | SPARCS | 1 | -- | -- | -- | -- | -- | -- | -- | -- | x |
| Haeffel et al. (2017) | Social Problem-Solving Training | 2 | -- | -- | x | -- | -- | x | -- | -- | -- |
| Haines et al. (2015) | Diversion pilot scheme | 1 | -- | -- | -- | -- | x | -- | -- | -- | -- |
| Hoogsteder et al. (2018) | Responsive Aggression Regulation Therapy (Re-ART) Outpatient | 1 | -- | -- | -- | -- | x | -- | -- | -- | -- |
| Hubble et al. (2015) | Computerized training in negative emotion recognition | 1 | -- | -- | -- | -- | x | -- | -- | -- | -- |
| Keiley (2007) | Multiple Family Group Intervention (MFGI) | 1 | -- | x | -- | -- | -- | -- | -- | -- | -- |
| Keiley et al. (2015) | Multiple Family Group Intervention (MFGI) | 1 | -- | x | -- | -- | -- | -- | -- | -- | -- |
| Krakow et al. (2001) | Image Rehearsal Therapy | 2 | -- | -- | -- | -- | -- | -- | x | -- | x |
| Lane (1993) | Attributional Retraining Program | 1 | -- | -- | -- | -- | -- | x | -- | -- | -- |
| Lindblom et al. (2017) | "A new direction" cognitive intervention program | 1 | -- | -- | -- | -- | x | -- | -- | -- | -- |
| MacMahon & Gross 1988 | Aerobic exercise program | 1 | -- | -- | -- | x | -- | -- | -- | -- | -- |
| Marrow et al. (2012) | TARGET | 5 | x | -- | -- | x | -- | x | x | -- | x |
| Olafson et al. (2018) | TGTC-A (youth program) + Think Trauma (staff training) | 3 | -- | -- | x | x | -- | x | -- | -- | -- |
| Ovaert et al. (2003) | Structured Group Therapy | 3 | x | -- | -- | x | -- | -- | -- | -- | x |
| Pomeroy et al. (2001) | Psychoeducation Group Therapy | 2 | -- | -- | -- | -- | -- | x | -- | -- | x |
| Raider et al. (2008) | SITCAP-ART | 2 | x | -- | -- | -- | -- | -- | -- | -- | x |
| Riggs Romaine (2018) | Juvenile Justice Anger Management for girls | 1 | -- | x | -- | -- | -- | -- | -- | -- | -- |
| Rivard (2003) | Sanctuary Model | 1 | -- | -- | -- | -- | -- | -- | x | -- | -- |
| Rohde et al. (2004) | Coping course | 2 | -- | -- | -- | x | -- | x | -- | -- | -- |
| Scheck et al. (1998) | Eye Movement Desensitization and Retraining (EMDR) | 1 | -- | -- | -- | -- | -- | -- | -- | -- | 1 |
| Seivert et al. (2014) | Animal Assisted Therapy | 1 | -- | -- | -- | -- | -- | 1 | -- | -- | 1 |
| Smith et al. (2012) | Multidimensional Treatment Foster Care Plus Trauma (MTFC + T) | 2 | X | -- | -- | -- | -- | -- | -- | -- | x |
| Strom et al. (2017) | Values-Based Therapeutic Environment (VBTE) | 1 | -- | -- | -- | -- | x | -- | -- | -- | -- |

Note: ^a^ x = primary study included in review. SITCAP-ART = Structured Sensory Intervention for Traumatized Children, Adolescents and Parents – Adjudicated and at-Risk Youth; SPARCS = Structured Psychotherapy for Adolescents Responding to Chronic Stress, TARGET = Trauma Affect Regulation: Guide for Education and Therapy; TF-CBT = Trauma-Focused Cognitive Behavioral Therapy; TGCT-A = Trauma and Grief Component Therapy-Adolescent; WRITE-ON = Writing and Reflecting on Identity To Empower Ourselves as Narrators.

**References for group-based primary studies included in systematic reviews and meta-analyses.**

Ahrens, J., & Rexford, L. (2002). Cognitive processing therapy for incarcerated adolescents with PTSD. *Journal of Aggression, Maltreatment & Trauma, 6*(1), 201–216. <https://doi.org/10.1300/J146v06n01_10>

Baetz, C. L., Branson, C. E., Weinberger, E., Rose, R. E., Petkova, E., Horwitz, S. M., & Hoagwood, K. E. (2022). The Effectiveness of PTSD Treatment for Adolescents in the Juvenile Justice System: A Systematic Review. *Psychological Trauma*, *14*(4), 642–652. <https://doi.org/10.1037/tra0001073>

Baglivio, M., & Jackowski, K. (2015). Evaluating the effectiveness of a victim impact intervention through the examination of changes in dynamic risk scores. *Criminal Justice Policy Review, 26*(1), 7–28. <https://doi.org/10.1177/0887403413489706>

Bahr, Cherrington, D. J., & Erickson, L. D. (2016). An evaluation of the impact of goal setting and cell phone calls on juvenile rearrests. *International Journal of Offender Therapy and Comparative Criminology, 60*(16), 1816–1835. <https://doi.org/10.1177/0306624X15588549>

Banks, B., Kuhn, T., Blackford, J. U. (2015). Modifying dialectical behavior therapy for incarcerated female youth: A pilot study. *OJJDP Journal of Juvenile Justice, 4*(1), 1-17.

Barnert, E. S., Himelstein, S., Herbert, S., Garcia-Romeu, A., & Chamberlain, L. J. (2014). Exploring an intensive meditation intervention for incarcerated youth. *Child and Adolescent Mental Health, 19*(1), 69–73. https://doi.org/10.1111/camh.12019

Bouffard, J. A. & Bergseth, K. J. (2008). The impact of re-entry services on juvenile offenders’ recidivism. *Youth Violence and Juvenile Justice*, *6*, 295–318. https ://doi.org/10.1177/00048 65812 46997 3.

Braboy, D. A. (2014). *The effectiveness of group intervention in reducing psychiatric symptoms in adolescent female offenders* (Doctoral Dissertation). ProQuest Dissertations Publishing.

Burraston, B. O., Bahr, S. J., & Cherrington, D. J. (2014). Reducing juvenile delinquency with automated cell phone calls. *International Journal of Offender Therapy and Comparative Criminology, 58*, 522–536. https ://doi.org/10.1177/03066 24X13 48094 7.

Caldwell, M. & Van Rybroek, G. J. (2001). Efficacy of a decompression treatment model in the clinical management of violent ouvenile offenders. *International Journal of Offender Therapy and Comparative Criminology, 45(*4), 469–477. <https://doi.org/10.1177/0306624X01454006>

Caldwell, M., Skeem, J., Salekin, R., & Van Rybroek, G. (2006). Treatment response of adolescent offenders with psychopathy features: A 2-year follow-up. *Criminal Justice and Behavior, 33*(5), 571–596. https://doi.org/10.1177/0093854806288176

Calleja, N. G. (2020). Assessing and Treating Trauma in Detained Adolescents: A Pre–Post Within Subjects Evaluation. *Journal of Child & Family Studies*, *29*(4), 934-941. <https://doi.org/10.1007/s10826-019-01564-9>

Cann J., Falshaw, L., & Friendship, C. (2005). Understanding “what works”: Accredited cognitive skills programmes for young offenders. *Youth Justice, 5*(3), 165–179. https://doi.org/10.1177/147322540500500303

Chamberlain, P., Leve, L. D., & DeGarmo, D. S. (2007). Multidimensional treatment foster care for girls in the juvenile justice system: 2-year follow-up of a randomized clinical trial. *Journal of Consulting and Clinical Psychology*, *75*(1), 187–193. https://doi.org/10.1037/0022-006X.75.1.187

Clair-Michaud, Martin, R. A., Stein, L. A. R., Bassett, S., Lebeau, R., & Golembeske, C. (2016). The impact of motivational interviewing on delinquent behaviors in incarcerated adolescents. *Journal of Substance Abuse Treatment, 65,* 13–19. https://doi.org/10.1016/j.jsat.2015.09.003

Cohen, J. A., Mannarino, A. P., Jankowski, K., Rosenberg, S., Kodya, S., & Wolford, G. L., II. (2016). A randomized implementation study of trauma-focused cognitive behavioral therapy for adjudicated teens in residential treatment facilities [Empirical Study; Quantitative Study]. *Child Maltreatment*, *21*(2), 156-167. <https://doi.org/https://dx.doi.org/10.1177/1077559515624775>

Day, A., Maddicks, R., McMahon, D. (1993). Brief prescriptive psychotherapy for depression with an incarcerated young offender model: An application of Barkham’s 2+1 model. *Journal of Offender Rehabilitation, 19*(1-2), 75–89. https://doi.org/10.1300/J076v19n01_05

Day, J. C., Zahn, M. A., & Tichavsky, L. P. (2015). What works for whom? The effects of gender responsive programming on girls and boys in secure detention*. The Journal of Research in Crime and Delinquency, 52*(1), 93–129. https://doi.org/10.1177/0022427814538033

Elwyn, L. J., Esaki, N., & Smith, C. A. (2015). Safety at a girls secure juvenile justice facility [Empirical Study; Interview; Quantitative Study]. *Therapeutic Communities*, *36*(4), 209-218. <https://doi.org/https://dx.doi.org/10.1108/TC-11-2014-0038>

Farrington, D. P., Ditchfield, J., Howard, P., & Jolliffe, D. (2002). Two intensive regimes for young offenders: a follow-up evaluation. *Findings, 163*, 1–4. Home Office (London, England)

Ford, J. D., & Hawke, J. (2012). Trauma affect regulation psychoeducation group and milieu intervention outcomes in juvenile detention facilities. *Journal of Aggression, Maltreatment & Trauma, 21*, 365–384. doi:10.1080/10926771.2012.673538

Ford, J. D., Steinberg, K. L., Hawke, J., Levine, J., & Zhang, W. (2012). Randomized trial comparison of emotion regulation and relational psychotherapies for PTSD with girls involved in delinquency. *Journal of Clinical Child & Adolescent Psychology, 41*, 27–37. doi:10.1080/15374416.2012.632343

Forgays, D. K., & DeMilio, L. (2005). Is Teen Court effective for repeat offenders? A test of the restorative justice approach. *International Journal of Offender Therapy & Comparative Criminology, 49*, 107–118. https ://doi.org/10.1177/03066 24X04 26941 1.

Greenbaum, C. A., & Javdani, S. (2017). Expressive writing intervention promotes resilience among juvenile justice-involved youth. *Children and Youth Services Review*, *73*, 220-229. https://doi.org/https://dx.doi.org/10.1016/j.childyouth.2016.11.034

Habib, M., Labruna, V., & Newman, J. (2013). Complex histories and complex presentations: Implementation of a manually-guided group treatment for traumatized adolescents. *Journal of Family Violence, 28*(7), 717–728. https://doi.org/10.1007/s10896-013-9532-y

Haeffel, G. J., Hein, S., Square, A., Macomber, D., Lee, M., Chapman, J., & Grigorendko, E. (2017). Evaluating a social problem solving intervention for juvenile detainees: Depressive outcomes and moderators of effectiveness. *Development and Psychopathology, 29*, 1035-1042. doi:10.1017/S0954579416001000

Haines, A., Lane, S., McGuire, J., Perkins, E., & Whittington, R. (2015). Offending outcomes of a mental health youth diversion pilot scheme in England. Criminal Behaviour and Mental Health, 25(2), 126–140. https://doi.org/10.1002/cbm.1916

Hoogsteder, L. M., Stams, G. J. J. ., Schippers, E. E., & Bonnes, D. (2018). Responsive Aggression Regulation Therapy (Re-ART): An evaluation study in a Dutch juvenile justice institution in terms of recidivism. *International Journal of Offender Therapy and Comparative Criminology, 62*(14), 4403–4424. https://doi.org/10.1177/0306624X18761267

Hubble, K., Bowen, K. L., Moore, S. C., & Van Goozen, S. H. M. (2015). Improving negative emotion recognition in young offenders reduces subsequent crime. *PloS One, 10*(6), e0132035–e0132035. <https://doi.org/10.1371/journal.pone.0132035>

Keiley, M. K. (2007). Multiple-family group intervention for incarcerated adolescents and their families: A pilot project. *Journal of Marital and Family Therapy, 33*(1), 106–124.

Keiley, M. K., Zaremba-Morgan, A., Datubo-Brown, C., Pyle, R., & Cox, M. (2015). Multiple-family group intervention for incarcerated male adolescents who sexually offend and their families: Change in maladaptive emotion regulation predicts adaptive change in adolescent behaviors. *Journal of Marital and Family Therapy, 41*(3), 324–339.

Krakow, B., Sandoval, D., Schrader, R., Keuhne, B., McBride, L., Yau, C. L., & Tandberg, D. (2001). Treatment of chronic nightmares in adjudicated adolescent girls in a residential facility. *Journal of Adolescent Health, 29*, 94-100

Lane, V. L. (1993). *Attributional retraining as an intervention strategy for incarcerated male adolescents* (Doctoral Dissertation). ProQuest Dissertations Publishing.

Lindblom, S., Eriksson, L., & Hiltunen, A. J. (2017). Evaluation of the cognitive intervention programme “A New Direction” targeting young offenders in Sweden. *Journal of Scandinavian Studies in Criminology and Crime Prevention, 18*(2), 176–190. https://doi.org/10.1080/14043858.2017.1307545

MacMahon, J. R., & Gross, R. T. (1988). Physical and psychological effects of aerobic exercise in delinquent adolescent males. *American Journal of Diseases of Children*, *142*(12), 1361–1366. <https://doi.org/10.1001/archpedi.1988.02150120115053>

Marrow, M. T., Knudsen, K. J., Olafson, E., & Bucher, S. E. (2012). The value of implementing TARGET within a trauma-Informed juvenile justice setting. *Journal of Child & Adolescent Trauma*, *5*(3), 257-270. <https://doi.org/10.1080/19361521.2012.697105>

Olafson, E., Boat, B. W., Putnam, K. T., Thieken, L., Marrow, M. T., & Putnam, F. W. (2018). Implementing Trauma and Grief Component Therapy for Adolescents and Think Trauma for Traumatized Youth in Secure Juvenile Justice Settings. *Journal of Interpersonal Violence*, *33(16)*, 2537-2557. https://doi.org/https://dx.doi.org/10.1177/0886260516628287

Ovaert, L. B., Cashel, M. L., * Sewell, K. W. (2003). Structured group therapy for posttraumatic stress disorder in incarcerated male juveniles. *American journal of orthopsychiatry, 73*(3), 294-301.

Pomeroy, E. C., Green, D. L., & Kiam, R. (2001). Female juvenile offenders incarcerated as adults: A psychoeducational group intervention. *Journal of Social Work, 1*(1), 101-115.

Raider, M. C., Steele, W., Delillo-Storey, M., Jacobs, J., & Kuban, C. (2008). Structured sensory therapy (SITCAP-ART) for traumatized adjudicated adolescents in residential treatment. *Residential Treatment for Children and Youth, 25*(2), 167-185. https://doi.org/10.1080/08865710802310178

Riggs Romaine, C. L., Kemp, K., Giallella, C. L., Goldstein, N. E., Serico, J., & Kelley, S. (2018). Can we hasten development? Effects of treatment on psychosocial maturity. *International Journal of Offender Therapy and Comparative Criminology, 62*(9), 2857–2876.

Rivard, J. C., Bloom, S. L., Abramovitz, M. D., Pasquale, L. E., Duncan, M., McCorkle, D., & Gelman, A. (2003). Assessing the implementation and effects of a trauma-focused intervention for youths in residential treatment. *Psychiatric Quarterly, 74*, 137-154.

Rohde, P., Jorgensen, J. S., Seeley, J. R., & Mace, D. E. (2004). A pilot evaluation of the coping course: A cognitive-behavioral intervention to enhance coping skills in incarcerated youth. *American Academy of Child and Adolescent Psychiatry, 43*, 669-676.

Scheck, M. M., Schaeffer, J. A., & Gillette, C. (1998). Brief psychological intervention with traumatized young women: The efficacy of eye movement desensitization and reprocessing. *Journal of Traumatic Stress, 11*(1), 25–44. https://doi.org/10.1023/A:1024400931106

Seivert, N. P., Cano, A., Casey, R. J., Johnson, A., & May, D. K. (2018). Animal assisted therapy for incarcerated youth: A randomized controlled trial. *Applied Developmental Science, 22*(2), 139–153. <https://doi.org/10.1080/10888691.2016.1234935>

Smith, D. K., Chamberlain, P., & Deblinger, E. (2012). Adapting multidimensional treatment foster care for the treatment of cooccurring trauma and delinquency in adolescent girls. *Journal of Child & Adolescent Trauma, 5*, 224–238. doi:10.1080/19361521.2012.697101

Strom, K. J., Hendrix, J. A., Dawes, D., & Anderson, S. H. (2017). An outcome evaluation of the Methodist Home for Children’s value-based therapeutic environment model. *Journal of Experimental Criminology, 13*(1), 101–124. https://doi.org/10.1007/s11292-016-9275-x
